# Different clinical features according to the anastomotic leakage subtypes after rectal cancer surgeries: contained vs. free leakages

**DOI:** 10.1371/journal.pone.0208572

**Published:** 2018-12-12

**Authors:** Eun Jung Park, Jeonghyun Kang, Hyuk Hur, Byung Soh Min, Seung Hyuk Baik, Kang Young Lee, Nam Kyu Kim

**Affiliations:** Division of Colon and Rectal Surgery, Department of Surgery, Yonsei University College of Medicine, Seoul, Korea; University of Bologna, ITALY

## Abstract

**Background:**

Anastomotic leakage can be classified by free and contained leakage according to clinical manifestations. The risk factors and their comparison between these leakage subtypes are uncertain. This study aims to evaluate anastomotic leakage patterns and to compare clinical features between free and contained leakages after low anterior resection for rectal cancer.

**Materials and methods:**

Between January 2005 and December 2012, a total of 2035 consecutive patients who underwent low anterior resection for primary rectal cancer were evaluated retrospectively at two-tertiary referral centers. The primary end points of this study were to assess detailed clinical features among leakage subtypes. The secondary end point was to compare risk factors between free and contained leakages.

**Results:**

Patients were subdivided into a no leakage group (n = 1890), free leakage group (n = 73), and contained leakage group (n = 72). Free leakage occurred more frequently in laparoscopic and robotic surgeries than open surgery (p = 0.015). On the other hand, contained leakage was developed in a higher rate of patients who received preoperative chemoradiotherapy (p<0.001). The mean development time was 4.6 days in the free leakage group, and 23.6 days in the contained leakage group. Patients with free leakage had a lower rate of a defunctioning stoma than contained leakage (5.5% vs. 29.2%, p<0.001). Risk factors for free leakage were smoking, tumor location, and laparoscopic surgery. However, tumor location and preoperative chemoradiotherapy increased the risk for contained leakage.

**Conclusions:**

Contained leakage in rectal cancer surgery showed different clinical manifestations and risk factors compared to free leakage. It is necessary to pay more attention to patients with particular risk factors for anastomotic leakage subtypes.

## Introduction

Anastomotic leakage is one of the most lethal complications after colorectal surgery. The reported incidence of anastomotic leakage is 1% to 19% following colorectal surgery, but is more common in rectal cancer surgery.[1, 2] Considerable efforts have been made to identify the factors associated with the prevention of anastomotic leakage, because it results in negative oncologic impact as well as delayed recovery.[3, 4] However, various arbitrary definitions of anastomotic leakage have become obstacles in the understanding of the overall characteristics and manifestations.[5]

Anastomotic leakage has been defined using different standards such as clinical symptoms, development time, grade of surgical intervention, and severity of anastomotic disruption. Floodeen et al. compared early and late symptomatic anastomotic leakage after low anterior resection according to the time of diagnosis.[6] In contrast, in 2010, the International Study Group of Rectal Cancer proposed a definition of anastomotic leakage based on severity grading for surgical intervention.[5] However, other studies described free and contained leakages. Free leakage presents with major anastomotic disruption with generalized peritonitis, while contained leakage presents as a minor anastomotic defect with localized peritonitis, including intra-abdominal abscess and fistula.[7–9] However, in this classification, it was difficult to immediately differentiate between contained and free leakages because the former could have subtle manifestations and was sometimes detected after discharge.[8]

Accordingly, most previous studies included contained leakage in the overall category of anastomotic leakage plus free leakage.[5, 10, 11] Moreover, delayed contained leakage, which was developed later than the inclusion criteria, was omitted in a study population of anastomotic leakage.[12] Until now, the clinical features of contained leakage are not fully understood in spite of its clinical significance. It is crucial to understand the detailed characteristics and their risk factors according to the anastomotic leakages.

Therefore, this study aimed to evaluate clinical manifestations and to compare risk factors for free and contained leakages after rectal cancer surgeries, using a precise definition of anastomotic leakage subtypes.

## Materials and methods

### Patient selection and data collection

**Fig 1:**
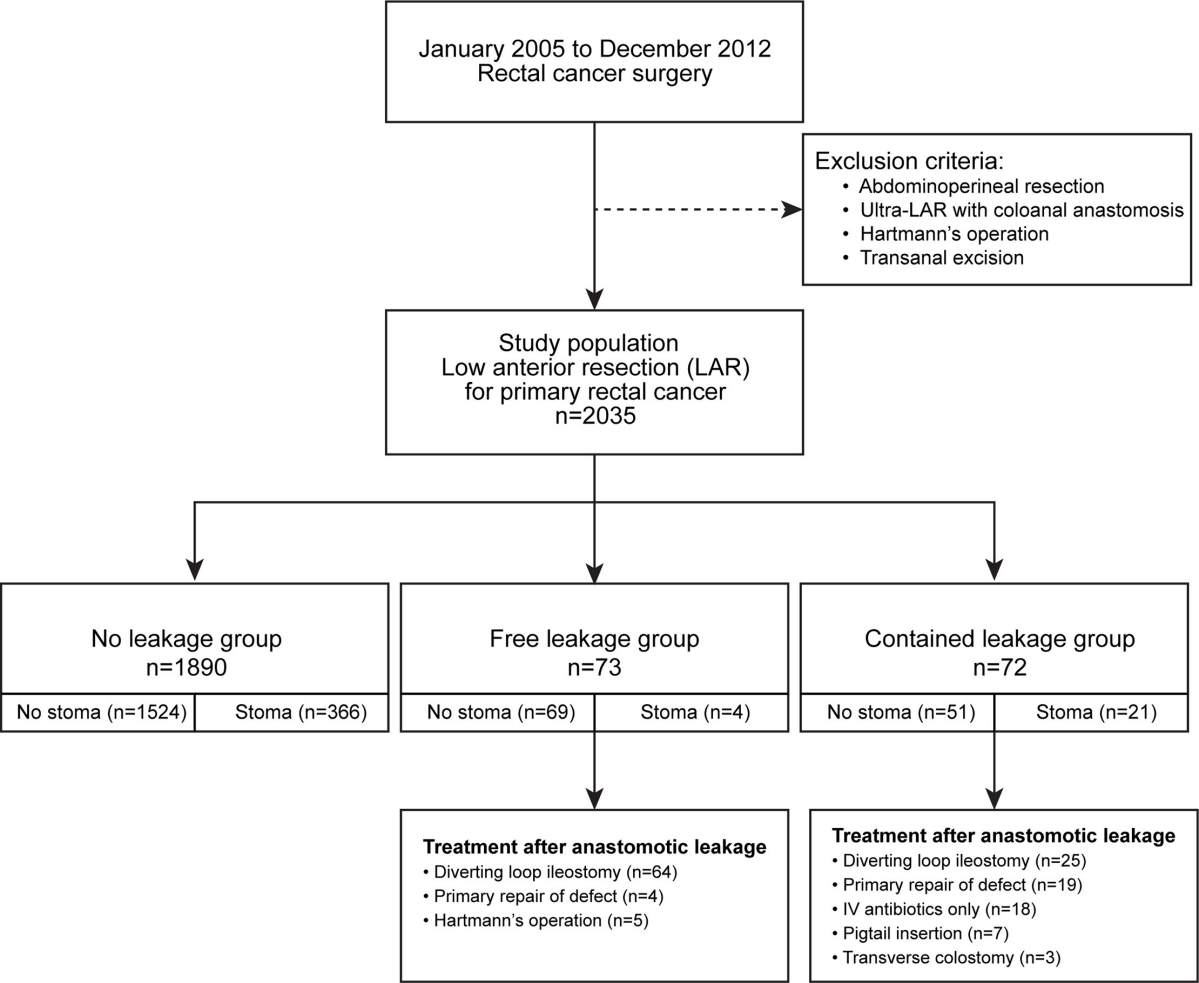
Flow chart of patient selection. LAR, Low anterior resection.

From January 2005 to December 2012, patients diagnosed with rectal cancer were evaluated in two institutions (Severance Hospital and Gangnam Severance Hospital) of the Yonsei University Health System, Seoul, South Korea. Of these, patients who underwent low anterior resection to treat primary rectal cancer in all stages were included. On the other hand, abdominoperineal resection, intersphincteric resection, Hartmann’s operation, and transanal excision were excluded. Finally, a total of 2035 consecutive patients were evaluated in this study. We divided these patients into three leakage groups according to the definition of anastomotic leakage as demonstrated in Fig 1: a no leakage group (n = 1890), a free leakage group (n = 73), and a contained leakage group (n = 72). Data were collected into the Yonsei Colorectal Cancer Database prospectively, and reviewed retrospectively. The Institutional Review Board of Gangnam Severance Hospital, Yonsei University, Seoul, Korea, approved this study according to good clinical practice guidelines and the principles of the Declaration of Helsinki (IRB No. 3-2016-0043). Informed consent was waived because this study was a retrospective study, which was approved by the institutional review board.

### Definition of anastomotic leakage

Anastomotic leakage is defined as a defect of the anastomotic site, which results in escape of bowel contents from the intraluminal to the extraluminal space of the intestine, according to the proposal of the International Study Group of Rectal Cancer.[5] For this study, we categorized anastomotic leakage as free leakage and contained leakage. Free leakage was defined as a major disruption of the anastomotic site, resulting in free perforation of the rectal wall with generalized peritonitis due to diffuse contamination of the abdomen by the bowel contents. Contained leakage was defined as a minor defect of the anastomotic site leading to limited contamination of the pelvic cavity, with localized peritonitis. The presence of rectovaginal fistula, rectovesical fistula, or perirectal abscess was included in contained leakage. Anastomotic leakage subtypes were categorized by reviewing all electronic medical charts and the results of cross-sectional imaging findings.

### Operative procedure and preoperative chemoradiotherapy

All surgical procedures were performed by nine surgeons, including four surgeons with more than 10 years of experience and five surgeons with less than 10 years’ experience. Laparoscopic surgery was performed using conventional laparoscopic instruments, and robotic surgery was performed with the da Vinci surgical system (Intuitive Surgical, Inc., Sunnyvale, CA, USA). All surgeries were performed according to the principles of tumor-specific mesorectal excision.[13] Anastomotic fashion was performed by the double-stapling method in all cases.

A defunctioning stoma was created selectively at the surgeon’s discretion during the operation if there were high risks of anastomotic leakage, such as positive findings on air insufflation, an incomplete donut after stapling, injury of the rectal wall during dissection, severe comorbid disease, or poor nutritional status of the patient.

Preoperative chemoradiotherapy (CRT) was performed for patients diagnosed with mid or low rectal cancer with T3-4 or positive lymph nodes in preoperative imaging studies, using 50.4 Gy as a total radiotherapy dose and 5-fluorouracil or capecitabine for 5 weeks. Surgery was performed within 6 to 8 weeks after completion of preoperative CRT.

### Definition of analyzed parameters

Hospital stay was estimated from the day of the operation to discharge. Readmission was defined when the patient was admitted again due to surgery-related complications after discharge. Pathologic outcomes followed the tumor-node-metastasis (TNM) staging system of the American Joint Committee on Cancer Manual, seventh edition.[14]

The development time of anastomotic leakage was measured from the operative day to the day of occurrence of anastomotic leakage. In this study, we did not limit the development period of anastomotic leakage, regardless of the time of admission and discharge. The diagnostic method was presented by the first diagnostic tool to detect anastomotic leakage. The mortality of this study was assessed within 30 postoperative days.

The grading of anastomotic leakage was categorized by the definition of the International Study Group of Rectal Cancer: Grade A required no active therapeutic intervention, Grade B required active therapeutic intervention without a re-laparotomy, and Grade C required a re-laparotomy.[5]

### Statistical analysis

Categorical variables were evaluated by the chi-square test or Fisher’s exact test. Continuous variables were analyzed by one-way analysis of variance among groups (ANOVA) and independent t-tests. Univariate and multivariate analysis for anastomotic leakage risk factors were analyzed by multinomial or binomial logistic regression models. The no leakage group was the reference category in both logistic regression models. Multivariate analysis was evaluated for risk factors, with a P value less than 0.05 in the univariate analysis. The odds ratio and 95% confidence intervals of multivariate analysis were schematized by a forest plot. Statistical analyses were performed using the SPSS program (Statistical Product and Service Solution 20 for Windows; SPSS Inc., Chicago, IL, USA) and Comprehensive Meta-Analysis statistical software (version 2.0; Biostat, Englewood, NJ, USA). A P value less than 0.05 was considered statistically significant.

## Results

### Patient characteristics

Patients of the free leakage group were younger than both the no leakage and the contained leakage group. Smoking history was greater in the free leakage than the no leakage group. On the other hand, contained leakage occurrence differed according to the tumor location compared to the no leakage group. Patients with upper rectal cancer comprised 30.5% of the no leakage group, which was larger than the 12.5% seen in the contained leakage group (p = 0.009). The difference between the no leakage and the free leakage group was marginal (p = 0.054). In surgical methods, free leakage occurred more frequently in laparoscopic and robotic surgeries compared to open surgery. Preoperative CRT was performed more frequently in the contained leakage than the no leakage group (44.4% vs. 19.9%, p <0.001). Preoperative albumin level in patients with contained leakage was lower than patients with no leakage. Other parameters did not show significant differences in leakage patterns (Table 1).

**Table 1:** Patient characteristics.

	No leakage	Free leakage	Contained leakage	p-value	p-value	p-value	p-value
	(n = 1890)	(n = 73)	(n = 72)		(No vs. Free)	(No vs. Contained)	(Free vs. Contained)
**Sex**				0.200†	0.225	1.000	0.780
Male	1178 (62.3%)	53 (72.6%)	46 (63.9%)				
Female	712 (37.7%)	20 (27.4%)	26 (36.1%)				
**Age** (year)	60.5±10.9 (22–87)	57.0±12.4 (31–82)	61.4±10.4 (34–83)	0.019*	**0.021**	1.000	**0.042**
**Weight** (kg)	62.0±10.0 (34.4–120.0)	64.4±12.1 (37.0–95.0)	62.2±10.7 (36.0–83.0)	0.140*	0.143	1.000	0.537
**BMI**	23.3±3.0 (14.7–38.1)	23.2±3.2 (17.0–35.0)	23.2±3.5 (16.5–34.7)	0.847*	1.000	1.000	1.000
**Smoking**				**0.002†**	**0.003**	0.474	0.624
Yes	611 (32.3%)	37 (50.7%)	29 (40.3%)				
No	1279 (67.7%)	36 (49.3%)	43 (59.7%)				
**DM**				0.535†	1.000	1.000	1.000
Yes	272 (14.4%)	8 (11.0%)	8 (11.1%)				
No	1618 (85.6%)	65 (89.0%)	64 (88.9%)				
**History of abdominal surgery**				0.581†	1.000	1.000	0.957
Yes	248 (13.1%)	8 (11.0%)	12 (16.7%)				
No	1642 (86.9%)	65 (89.0%)	60 (83.3%)				
**ASA**				0.763††	1.000	1.000	1.000
1	843 (44.6%)	38 (52.1%)	31 (43.1%)				
2	979 (51.8%)	33 (45.2%)	38 (52.8%)				
3	68 (3.6%)	2 (2.7%)	3 (4.2%)				
**Tumor location**				**0.001**†	0.054	**0.009**	1.000
High (10.1-15cm)	577 (30.5%)	14 (19.2%)	9 (12.5%)				
Mid (5.1-10cm)	1084 (57.4%)	54 (74.0%)	55 (76.4%)				
Low (0-5cm)	229 (12.1%)	5 (6.8%)	8 (11.1%)				
**Surgeon's experience**				0.337†	1.000	0.477	0.612
>10 year	1254 (66.3%)	50 (68.5%)	42 (58.3%)				
≤10 year	636 (33.7%)	23 (31.5%)	30 (41.7%)				
**Surgical method**				**0.007**†	**0.015**	0.402	1.000
Open	744 (39.4%)	16 (21.9%)	20 (27.8%)				
Laparoscopy	660 (34.9%)	37 (50.7%)	31 (43.1%)				
Robot	486 (25.7%)	20 (27.4%)	21 (29.2%)				
**Preoperative CRT**				**<0.001**†	0.975	**<0.001**	0.036
Yes	377 (19.9%)	18 (24.7%)	32 (44.4%)				
No	1513 (80.1%)	55 (75.3%)	40 (55.6%)				
**Preoperative Hb** (g/dL)	13.0±1.7 (7.0–18.0)	13.2±1.6 (10.0–17.0)	12.6±1.6 (8.0–16.0)	0.090*	1.000	0.110	0.158
**Preoperative albumin** (g/dL)	4.3±0.4 (2.3–5.5)	4.3±0.4 (3.0–5.2)	4.2±0.5 (2.6–5.1)	0.054*	1.000	**0.048**	0.320

n (%); Mean ± standard deviation (range)

† Chi-square test

†† Fisher’s exact test

*ANOVA, BMI, body mass index; DM, diabetes mellitus; ASA, American Society of Anesthesiologists; CRT, chemoradiotherapy; Preop, preoperative; Hb, hemoglobin

### Perioperative and pathologic outcomes

Mean operative time in the contained leakage group was longer than the no leakage group. Intraoperative blood loss of the contained leakage group was larger than both the no leakage and the free leakage groups. However, the incidence of intraoperative transfusion, conversion, and combined resection was not significantly different among all the leakage groups. A defunctioning stoma was created in 5.5% of the free leakage group. This was a lower rate than the 19.4% in the no leakage group and 29.2% in the contained leakage group. The duration of hospital stay was longer in the free leakage group and the rate of readmission was higher in the contained leakage group than the other leakage groups. On the other hand, TNM stage, T stage, N stage, and histologic differentiation did not differ among the leakage groups (Table 2).

**Table 2:** Perioperative and pathologic outcomes according to anastomotic leakage subtypes.

	No leakage	Free leakage	Contained leakage	p-value	p-value	p-value	p-value
	(n = 1890)	(n = 73)	(n = 72)		(No vs. Free)	(No vs. Contained)	(Free vs. Contained)
**Operative time** (min)	257.8±99.6 (68–860)	276.1±104.7 (131–572)	291.7±111.8 (98–663)	0.007*	0.375	**0.015**	1.000
**Blood loss amount** (ml)	222.0±400.4 (0–6300)	144.5±237.2 (0–1000)	340.1±465.9 (0–3000)	0.011*	0.309	**0.041**	**0.009**
**Transfusion**				0.645††	1.000	1.000	1.000
Yes	93 (4.9%)	3 (4.1%)	5 (6.9%)				
No	1797 (95.1%)	70 (95.9%)	67 (93.1%)				
**Conversion**				0.393††	1.000	1.000	1.000
Yes	31 (1.6%)	2 (2.7%)	0 (0.0%)				
No	1859 (98.4%)	71 (97.3%)	72 (100.0%)				
**Combined resection**				0.397††	1.000	1.000	0.498
Yes	112 (5.9%)	2 (2.7%)	6 (8.3%)				
No	1778 (94.1%)	71 (97.3%)	66 (91.7%)				
**Defunctioning stoma**				**0.001**†	**0.009**	0.120	**<0.001**
Yes	366 (19.4%)	4 (5.5%)	21 (29.2%)				
No	1524 (80.6%)	69 (94.5%)	51 (70.8%)				
**Hospital stay** (day)	12.0±7.4 (3–91)	25.1±13.4 (8–73)	19.6±19.6 (6–121)	<0.001*	**<0.001**	**<0.001**	**<0.001**
**Readmission**				<0.001*	1.000	**<0.001**	**<0.001**
Yes	36 (1.9%)	2 (2.7%)	42 (58.3%)				
No	1854 (98.1%)	71 (97.3%)	30 (41.7%)				
**TNM stage**				0.522†	1.000	1.000	1.000
Stage O, pCR	71 (3.8%)	1 (1.4%)	4 (5.6%)				
Stage I	553 (29.3%)	16 (21.9%)	17 (23.6%)				
Stage II	489 (25.9%)	21 (28.8%)	15 (20.8%)				
Stage III	604 (32.0%)	26 (35.6%)	27 (37.5%)				
Stage IV	173 (9.2%)	9 (12.3%)	9 (12.5%)				
**T stage**				0.580††	1.000	1.000	1.000
Tis, T0	79 (4.2%)	1 (1.4%)	4 (5.6%)				
T1	284 (15.0%)	8 (11.0%)	6 (8.3%)				
T2	411 (21.7%)	14 (19.2%)	16 (22.2%)				
T3	1024 (54.2%)	46 (63.0%)	44 (61.1%)				
T4	92 (4.9%)	4 (5.5%)	2 (2.8%)				
**N stage**				0.216	1.000	0.243	1.000
N0	1163 (61.5%)	41 (56.2%)	37 (51.4%)				
N1	463 (24.5%)	21 (28.8%)	26 (36.1%)				
N2	264 (14.0%)	11 (15.1%)	9 (12.5%)				
**Histologic grade**				0.203††	1.000	0.480	1.000
Well	333 (17.6%)	8 (11.0%)	13 (18.1%)				
Moderate	1476 (78.1%)	61 (83.6%)	52 (72.2%)				
Poor	40 (2.1%)	3 (4.1%)	4 (5.6%)				
Mucinous	38 (2.0%)	1 (1.4%)	3 (4.2%)				
Signet-ring cell	3 (0.2%)	0 (0.0%)	0 (0.0%)				

n (%); Mean ± standard deviation (range)

^†^ Chi-square test

^††^ Fisher’s exact test

*ANOVA; CR, complete remission

### Comparison of clinical features between free and contained leakages

**Fig 2:**
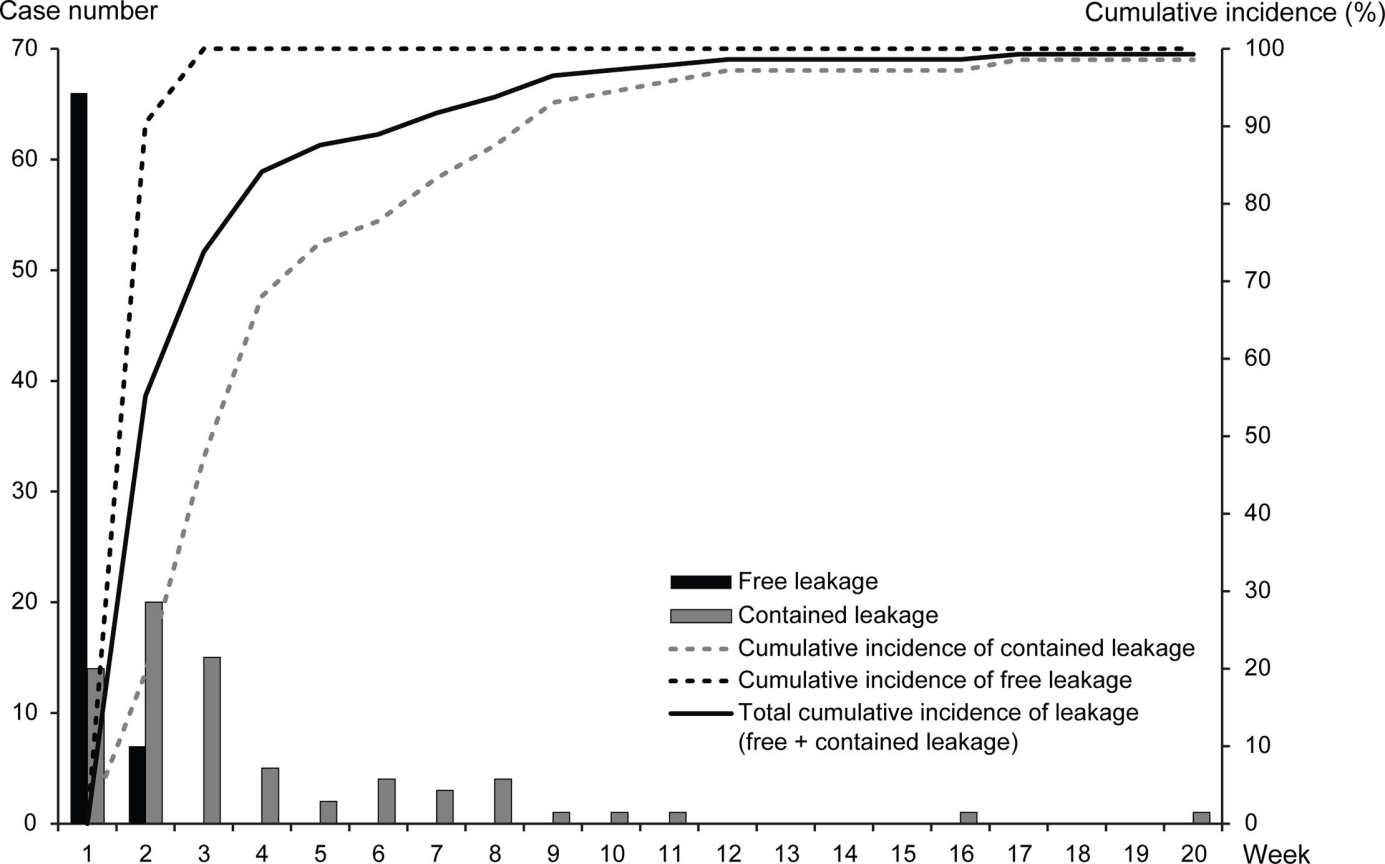
Incidence of anastomotic leakage.

The overall incidence of anastomotic leakage, including free and contained leakage, was 7.1%. Free leakage occurred in 73 patients (3.6%), and contained leakage in 72 patients (3.5%). As shown in Table 3, all free leakage occurred within 30 days after surgery, while 76.4% of contained leakage occurred within 30 postoperative days (p<0.001). The median development time of anastomotic leakage was 4.0 days in the free leakage group, and 15.5 days in the contained leakage group. In addition, the occurrence time for anastomotic leakage showed different patterns for free and contained leakages, as shown in Fig 2. Contained leakage occurred over a wide distribution range up until 20 postoperative weeks. However, all cases with free leakage developed within 2 postoperative weeks.

**Table 3:** Comparison of clinical features between free and contained leakages.

	Free leakage (n = 73)	Contained leakage (n = 72)	p-value
**Anastomotic leakage (Duration)**			**<0.001**^**†**^
Early period(≤30 days)	73 (100.0%)	55 (76.4%)	
Delayed period (>30 days)	0 (0.0%)	17 (23.6%)	
**Development time of anastomotic leakage from the initial operation (day)**	4.0 (3.0–6.0)*	15.5 (8.3–29.3)*	**<0.001**^**‡**^
**Anastomotic leakage (Grade-intervention)**^**¶**^			**<0.001**^**††**^
Grade A (no intervention)	0 (0.0%)	12 (16.7%)	
Grade B (intervention, but no re-laparotomy)	0 (0.0%)	6 (8.3%)	
Grade C (re-laparotomy)	73 (100.0%)	54 (75.0%)	
**Initial manifestations to detect anastomotic leakages**			**<0.001**^**††**^
Fever/ Abdominal pain/ leukocytosis	38 (52.0%)	26 (36.1%)	
Hypotension (+/- mental change)	7 (9.6%)	1 (1.4%)	
Color change of drainage	23 (31.5%)	8 (11.1%)	
Leukocytosis / leukocytopenia only	3 (4.1%)	6 (8.3%)	
Fecal discharge to vagina	0 (0.0%)	12 (16.7%)	
Fecal discharge to urethra	0 (0.0%)	1 (1.4%)	
Intestinal obstruction	1 (1.4%)	4 (5.6%)	
Perianal pain	1 (1.4%)	11 (15.2%)	
Scrotal swelling	0 (0.0%)	3 (4.2%)	
**Features of anastomotic leakage**			**<0.001**^**††**^
Anastomotic site disruption (major: defect ≥1cm)	55 (75.3%)	12 (16.7%)	
Anastomotic site disruption (minor: defect<1cm)	12 (16.4%)	24 (33.3%)	
Perianal/ pelvic abscess formation	4 (5.5%)	15 (20.8%)	
Anastomotic site necrosis	2 (2.7%)	0 (0.0%)	
Fistula formation in perirectal space	0 (0.0%)	7 (9.7%)	
Rectovaginal/ rectovesical fistula	0 (0.0%)	14 (19.4%)	
**Treatments after anastomotic leakage**			**<0.001**^**††**^
Diverting loop ileostomy	64 (87.7%)	25 (34.7%)	
Primary repair of anastomotic defect	4 (5.5%)	19 (26.4%)	
Transverse colostomy	0 (0.0%)	3 (4.2%)	
Hartmann's operation	5 (6.8%)	0 (0.0%)	
Pigtail insertion	0 (0.0%)	7 (9.7%)	
Use of IV antibiotics	0 (0.0%)	18 (25.0%)	
**Mortality within postoperative 30 days**	0 (0.0%)	1 (1.4%)	**0.497**^**††**^

¶The grade of anastomotic leakage was taken from the definition given by the International Study Group of Rectal Cancer

IV, intravenous; n (%); Mean ± standard deviation (range)

^†^ Chi-square test

^††^ Fisher’s exact test

‡ Independent t-test

*,median value (interquartile range)

Initial clinical manifestations to detect anastomotic leakages showed different patterns in the free and contained leakage groups (p<0.001). Abdominal pain combined with high fever and leukocytosis was the most common clues to detect both free and contained leakages before radiologic evaluation. However, serious clinical manifestations, such as hypotension and mental status change occurred as a higher rate in the free leakage than the contained leakage group. On the other hand, various nonspecific symptoms of anastomotic leakage such as perianal pain, scrotal swelling, and intestinal obstruction were found more frequently in the contained leakage than the free leakage group. Perirectal, rectovaginal, or rectovesical fistulas occurred only in the contained leakage group.

All patients with free leakage were Grade C and required a re-laparotomy. However, 75% of patients with contained leakage were Grade C, 16.7% Grade A, and 8.3% Grade B (p<0.001). The most common cause of free leakage was anastomotic site disruption with a defect greater than 1 cm. However, anastomotic site disruption with a less than 1 cm defect was the major cause of contained leakage.

In the treatment after anastomotic leakage, diverting loop ileostomy was the most common treatment in both leakage groups, and was performed more frequently in the free leakage than contained leakage group (87.7% vs. 34.7%). Conversely, primary repair of the anastomotic site defect was performed more frequently in the contained leakage group (5.5% vs. 26.4%).

Mortality due to progression of sepsis within 30 postoperative days occurred in one patient (1.4%) in the contained leakage group. On the other hand, there was no mortality in the free leakage group. (Table 3)

### Risk factors for anastomotic leakage

Risk factors for anastomotic leakage were different between free and contained leakages by multinomial logistic regression results. In univariate analysis, smoking history increased the risk of free leakage. Mid-rectal cancer increased the risk of free and contained leakages. Although the risk for free leakage was 2.61 times greater for laparoscopic surgery and 1.91 times for robotic surgery, contained leakage was not affected by the surgical method. Preoperative CRT was not a risk factor for free leakage but was a risk factor for contained leakage. While a defunctioning stoma decreased the risk of free leakage (OR = 0.24, p = 0.006), it did not decrease the risk of contained leakage (OR = 1.72, p = 0.042). Preoperative albumin level less than 3.3 g/dL was a risk factor for contained leakage. Additionally, we evaluated risk factors for total leakage compared with no leakage by binomial logistic regression. Because we intended to compare our results with previous studies using a mixed definition including free and contained leakages, we evaluated the total leakages in this study. Smoking, tumor location, surgical method, and preoperative CRT were risk factors for total leakage. The results were similar for free leakage except for preoperative CRT. (Table 4)

**Table 4:** Univariate analysis of risk factors for anastomotic leakage by the multinomial logistic regression.

	p-value^†^	Free leakage^†^		Contained leakage^†^		Total leakage (free & contained)^††^	
		OR ratio (95% CI for Exp β)	p-value^†^	OR ratio (95% CI for Exp β)	p-value^†^	OR ratio (95% CI for Exp β)	p-value^††^
**Sex**							
Male vs. Female	0.187	1.60 (0.95–2.70)	0.077	1.07 (0.66–1.75)	0.788	1.30 (0.91–1.87)	0.154
**Age (year)**							
≥70 vs. <70	0.449	0.73 (0.40–1.35)	0.319	1.21 (0.71–2.07)	0.478	0.96 (0.64–1.44)	0.839
**BMI (kg/m**^**2**^**)**							
≥25 vs. <25	0.995	1.01 (0.60–1.70)	0.978	1.03 (0.61–1.74)	0.921	1.02 (0.70–1.48)	0.930
**Smoking**							
Yes vs. No	**0.003**	2.15 (1.35–3.44)	**0.001**	1.41 (0.87–2.28)	0.160	1.75 (1.24–2.46)	**0.001**
**DM**							
Yes vs. No	0.514	0.73 (0.35–1.54)	0.412	0.74 (0.35–1.57)	0.436	0.74 (0.43–1.26)	0.265
**Abdominal op history**							
Yes vs. No	0.591	0.82 (0.39–1.72)	0.591	1.32 (0.70–2.50)	0.385	1.06 (0.65–1.73)	0.818
**ASA**	0.783						0.785
2 vs. 1		0.75 (0.47–1.20)	0.231	1.06 (0.65–1.71)	0.827	0.89 (0.63–1.25)	0.490
3 vs. 1		0.65 (0.15–2.76)	0.562	1.20 (0.36–4.03)	0.768	0.90 (0.35–2.30)	0.823
**Tumor location**	**<0.001**						**<0.001**^**††**^
Mid vs. high		2.05 (1.13–3.73)	**0.018**	3.25 (1.60–6.63)	**0.001**	2.52 (1.59–4.00)	**<0.001**
Low vs. high		0.90 (0.32–2.53)	0.841	2.24 (0.85–5.88)	0.101	1.42 (0.71–2.86)	0.320
**Surgeon's experience**							
≤10 year vs. >10 year	0.347	0.91 (0.55–1.50)	0.704	1.41 (0.87–2.27)	0.160	1.14 (0.80–1.61)	0.477
**Surgical method**	**0.005**						**0.002**^**††**^
Laparoscopy vs. open		2.61 (1.44–4.73)	**0.002**	1.75 (0.99–3.10)	0.056	2.13 (1.40–3.23)	**<0.001**
Robot vs. open		1.91 (0.98–3.73)	0.057	1.61 (0.86–3.00)	0.135	1.74 (1.10–2.77)	**0.018**
Robot vs. laparoscopy		0.73 (0.42–1.27)	0.267	0.91 (0.52–1.61)	0.757	0.81 (0.54–1.22)	0.319
**Preoperative CRT**							
Yes vs. No	**<0.001**	1.31 (0.76–2.26)	0.326	3.21 (1.99–5.18)	**<0.001**	2.11 (1.47–3.03)	**<0.001**
**Operation time**							
≥4hr vs. <4hr	0.196	0.96 (0.60–1.53)	0.853	1.55 (0.95–2.50)	0.077	1.21 (0.86–1.70)	0.271
**Defunctioning stoma**							
Yes vs. No	**<0.001**	0.24 (0.09–0.67)	**0.006**	1.72 (1.02–2.89)	**0.042**	0.87 (0.56–1.36)	0.532
**Combined resection**							
Yes vs. No	0.315	0.45 (0.11–1.85)	0.266	1.44 (0.61–3.40)	0.402	0.93 (0.44–1.94)	0.840
**Conversion**							
Yes vs. No	0.243	1.69 (0.40–7.20)	0.478	<000.1 (5.2z10^-9^–5.2x10^-9^)	-	0.84 (0.20–3.54)	0.811
**Transfusion**							
Yes vs. No	0.718	0.83 (0.26–2.68)	0.753	1.44 (0.57–3.66)	0.442	1.13 (0.54–2.37)	0.750
**TNM stage**							
Stage III-IV vs. stage I-II	0.179	1.32 (0.83–2.11)	0.246	1.43 (0.89–2.29)	0.135	1.37 (0.98–1.93)	0.065
**T stage**							
≥T3 vs. <T3	0.195	1.51 (0.91–2.49)	0.109	1.23 (0.75–2.00)	0.413	1.36 (0.95–1.94)	0.092
**Preoperative Hb (g/dL)**							
<12 vs. ≥12	0.774	1.02 (0.54–1.91)	0.956	1.25 (0.69–2.27)	0.465	1.13 (0.73–1.76)	0.586
**Preoperative albumin (g/dL)**							
<3.3 vs. ≥3.3	0.062	0.70 (0.09–5.14)	0.722	3.74 (1.42–9.81)	**0.007**	2.16 (0.90–5.21)	0.086

No leakage group is the reference category

^†^ Multinomial logistic regression (free leakage vs. no leakage; contained leakage vs. no leakage)

^††^ Binomial logistic regression (Total leakage vs. no leakage); OR, odds ratio; CI, confidence interval; BMI, body mass index; DM, diabetes mellitus; op, operation; ASA, American Society of Anesthesiologists; CRT, chemoradiotherapy; Hb, hemoglobin

**Fig 3:**
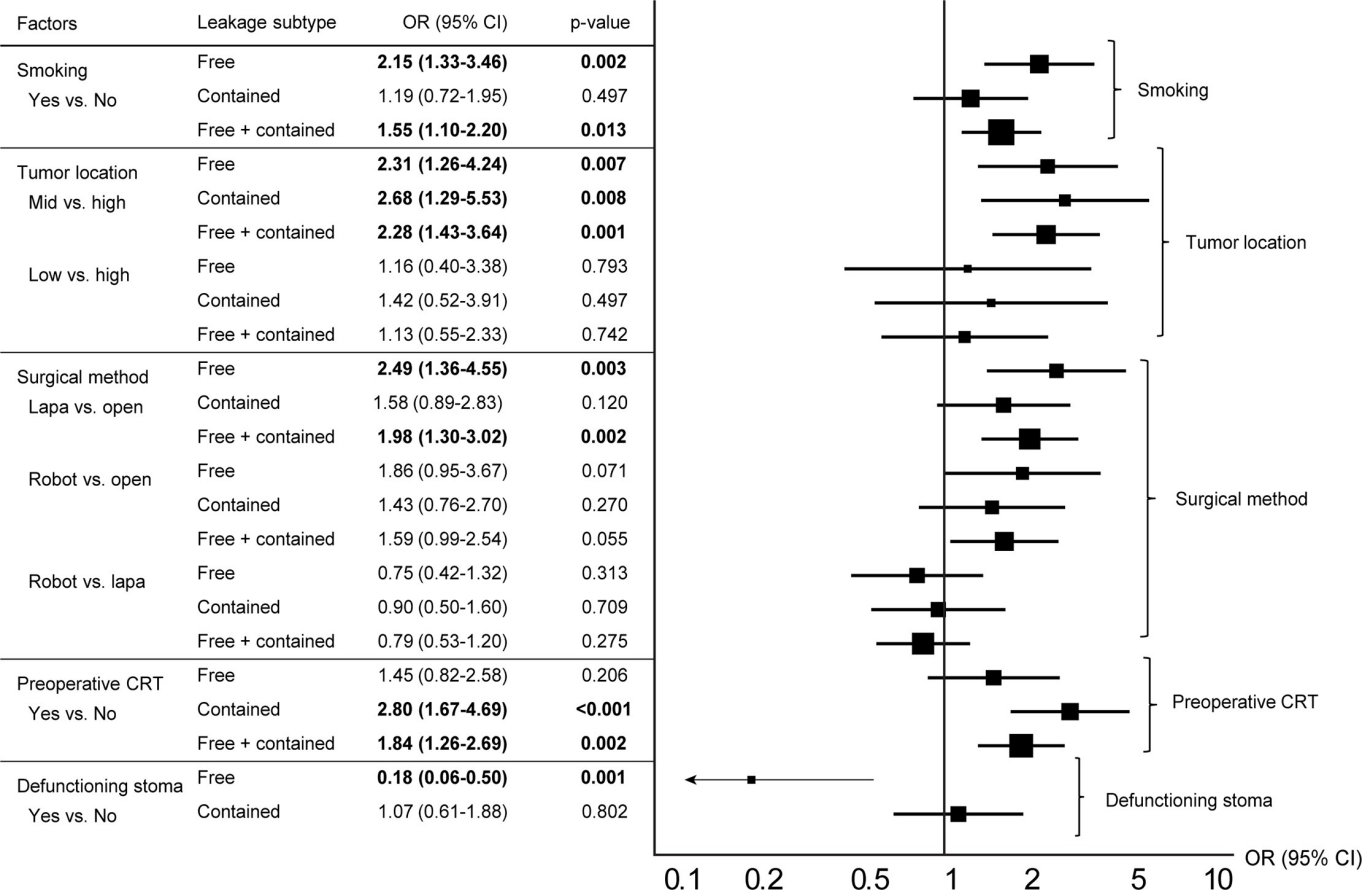
Forest plot for risk factors of anastomotic leakage subtypes. OR, odds ratio; CI, confidence interval; Lapa, laparoscopy; CRT, chemoradiotherapy.

In multivariate analysis, smoking history, tumor location, and laparoscopic surgery were risk factors for free leakage. However, a defunctioning stoma decreased the risk of free leakage (OR = 0.18, p = 0.001). Contained leakage was affected by tumor location and preoperative CRT. Preoperative CRT increased the risk of contained leakage by 2.80 times. Total leakage increased in patients with smoking history, mid-rectal cancer, and laparoscopic surgery according to binomial logistic regression. Preoperative CRT was also a risk factor for total leakage as well as contained leakage.(Table 5) These results of multivariate analysis are summarized by the forest plot in Fig 3.

**Table 5:** Multivariate analysis of risk factors for anastomotic leakage.

	p-value^†^	Free leakage^†^		Contained leakage^†^		Total leakage (free & contained)^††^	
		OR ratio (95% CI for Exp β)	p-value^†^	OR ratio (95% CI for Exp β)	p-value^†^	OR ratio (95% CI for Exp β)	p-value^††^
**Smoking**	**0.007**						
Yes vs. No		2.15 (1.33–3.46)	**0.002**	1.19 (0.72–1.95)	0.497	1.55 (1.10–2.20)	**0.013**
**Tumor location**	**0.001**						
Mid vs. high		2.31 (1.26–4.24)	**0.007**	2.68 (1.29–5.53)	**0.008**	2.28 (1.43–3.64)	**0.001**
Low vs. high		1.16 (0.40–3.38)	0.793	1.42 (0.52–3.91)	0.497	1.13 (0.55–2.33)	0.742
**Surgical method**	**0.019**						
Laparoscopy vs. open		2.49 (1.36–4.55)	**0.003**	1.58 (0.89–2.83)	0.120	1.98 (1.30–3.02)	**0.002**
Robot vs. open		1.86 (0.95–3.67)	0.071	1.43 (0.76–2.70)	0.270	1.59 (0.99–2.54)	0.055
Robot vs. laparoscopy		0.75 (0.42–1.32)	0.313	0.90 (0.50–1.60)	0.709	0.79 (0.53–1.20)	0.275
**Preoperative CRT**	**<0.001**						
Yes vs. No		1.45 (0.82–2.58)	0.206	2.80 (1.67–4.69)	**<0.001**	1.84 (1.26–2.69)	**0.002**
**Defunctioning stoma**	**<0.001**						
Yes vs. No		0.18 (0.06–0.50)	**0.001**	1.07 (0.61–1.88)	0.802	-	-

No leakage group is the reference category

^†^ Multinomial logistic regression

^††^ Binomial logistic regression; OR, odds ratio; CI, confidence interval; CRT, chemoradiotherapy

## Discussion

This study evaluated anastomotic leakage after low anterior resection for rectal cancer according to the leakage subtypes classified as free and contained leakages. The principal finding of this study was that clinical features and risk factors were different for free and contained leakages.

Initial clinical symptoms of free leakage presented with urgent symptoms related to acute systemic inflammation. On the other hand, contained leakage occurred with ambiguous symptoms such as perianal pain, scrotal swelling, or intestinal obstruction, which made it difficult to promptly identify anastomotic leakage. Moreover, while free leakage was diagnosed within two weeks in all patients, contained leakage was detected later and at different times, as shown in Fig 2, with a higher rate of readmission than free leakage. These findings suggest that free leakage tends to be detected immediately on initial admission. However, contained leakage may be diagnosed after readmission of patients with symptoms suspicious for anastomotic leakage, which was not found during the initial hospital stay after rectal cancer surgery. In the study of Damrauer et al., free leaks tended to be diagnosed during primary admission, and contained leaks presented equally before and after discharge.[8] In addition, Hyman et al. pointed out the underestimation of true anastomotic leakage, because anastomotic leaks with uncertain symptoms escaped from detection and were captured later discharge.[15] Previous studies support the clinical characteristics of contained leakage seen in this study. Subtle clinical manifestations and delayed occurrence of contained leakage tended to make it difficult to detect anastomotic leakage.

In this study, treatment after anastomotic leakage differed for free and contained leakages. All patients with free leakage required an immediate re-laparotomy to resolve anastomotic leakage. Meanwhile, patients with contained leakage were treated by non-surgical methods as well as a re-laparotomy. Relatively mild symptoms of contained leakage, which was limited to the pelvic cavity, may influence these results.

Anastomotic leakage risk factors were different according to leakage subtypes. Smoking history, tumor location, and laparoscopic surgery were risk factors for free leakage. Meanwhile, tumor location and preoperative CRT were risk factors for contained leakage. Interestingly, preoperative CRT was a risk factor in patients with contained leakage, but not in patients with free leakage. In fact, the effect of preoperative CRT for anastomotic leakage is still debatable in rectal cancer surgery. Some previous studies asserted that there was no influence of preoperative CRT on anastomotic leakage.[16–18] However, many papers support the negative effect of preoperative CRT on increases in anastomotic leakage rates.[19–21] In our results, preoperative CRT increased the risk of contained leakage, but did not affect free leakage. Because preoperative CRT induces an impaired healing process by degradation of irradiated tissues, contained leakage, which is induced by the chronic inflammatory response of anastomotic site defects, could be influenced more by radiotherapy than free leakage.

Although most previous studies stated that low rectal cancer has an increased risk of anastomotic leakage,[1, 22–24] this present study showed that the risk of free and contained anastomotic leakages was only seen in mid-rectal cancer. This result can be interpreted as the effect of the defunctioning stoma in low rectal cancer. In a systematic review and meta-analysis, the defunctioning stoma in low rectal cancer surgery is known to reduce the rate of relevant anastomotic leakage.[1, 25] In this study, a defunctioning stoma was created in 7.3% of upper, 21.4% of mid-, and 38.0% of low rectal cancer cases. Although it is known that patients with low rectal cancer have a higher risk of anastomotic leakage, an increased rate of a defunctioning stoma creation in low rectal cancer might compensate for the high risk of anastomotic leakage. In this study, a defunctioning stoma decreased the risk of free leakage (OR = 0.18, p = 0.001), while it had no effect on contained leakage (OR = 1.07, p = 0.802). Because a diverting stoma can reduce the acute inflammatory reaction caused by anastomotic disruption, through movement of fecal material to the outside of the abdomen, it might decrease the risk of free anastomotic leakage. Randomized clinical trial results for a defunctioning stoma correlated with our results for free leakage.[26] Meanwhile, the chronic inflammatory response caused by stagnant, contaminated contained leakage cannot be resolved by a defunctioning stoma formation. McDermott et al. suggested that the defunctioning stoma did not affect in preventing anastomotic leakage, but in decreasing the sequelae after leakage. This conclusion implies that a defunctioning stoma reduces free leakage, and but does not reduce contained leakage, according to our study results.

In terms of the surgical method, both laparoscopic and robotic surgeries had a greater risk of anastomotic leakage than open surgery for both free and total leakages in this study. According to a multicenter analysis by the Korean Laparoscopic Colorectal Study Group, laparoscopic surgery had drawbacks of anastomotic leakage with respect to a limited rectal transection, and difficulties using linear staplers in the narrow pelvic cavity.[20] In a case-matched study of mid- and low rectal cancer, anastomotic leakage occurred more frequently in laparoscopic and robotic surgeries compared with open surgery.[27] These findings are correlated with the results of free leakage. In these regards, surgeons need to consider open rectal cancer surgeries, including diversion of defunctioning stoma, for patients with high risks of anastomotic leakage, especially free leakage. Meanwhile, contained leakage was not affected by surgical methods in this study. It seems to be less influenced by the surgical method.

This study has inherent limitations owing to its retrospective study design. In addition, in the issue of the ostomy formation, the creation of a defunctioning stoma depends on the surgeon’s discretion. These limitations may bias the proper interpretation of the results in this study. However, we endeavored to understand anastomotic leakage after rectal cancer surgery to compare free and contained leakages with analyzing large numbers of study population. A meticulous analyses of leakage subtypes can provide a deeper understanding of anastomotic leakage, because previous studies generally focused only on acute, symptomatic anastomotic leakage, which is considered as free leakage.

## Conclusions

Free leakage is regarded as anastomotic leakage conventionally. However, our findings suggest that contained leakage had unique clinical features and different risk factors compared to free leakage. In particular, patients who received preoperative chemoradiotherapy are required to pay more attention to contained leakage although they have defunctioning stoma, which can reduce the risk of free leakage. Therefore, clinicians should consider these detailed characteristics of anastomotic leakage subtypes when treating patients with rectal cancer surgeries.

## Supporting information

S1 Data Raw data of patients is provided online. (XLSX)

## Abbreviations

CRT: Chemoradiotherapy
TNM: Tumor-node-metastasis
CT: Computed tomography
MRI: Magnetic resonance imaging
OR: Odds ratio
